# Enhancing the Textural Properties of Tibetan Pig Sausages via *Zanthoxylum bungeanum* Aqueous Extract: Polyphenol-Mediated Quality Improvements

**DOI:** 10.3390/foods14091639

**Published:** 2025-05-07

**Authors:** Jingjing Huang, Haiqiu Wei, Zhang Luo, Liang Li, Zhendong Liu, Ningning Xie

**Affiliations:** 1 Institute of Agro-Product Science and Technology, Anhui Academy of Agricultural Sciences, Hefei 230031, China; jingjingcau@163.com (J.H.); haiqiu.wei@a-owen.com (H.W.); 2 Anhui Engineering Laboratory of Food Microbial Fermentation and Functional Application, Hefei 230031, China; 3 Food Science College, Tibet Agriculture & Animal Husbandry University, Nyingchi 860000, China; luozhang1759@sohu.com (Z.L.); jwllok@sina.com (L.L.); liu304418091@126.com (Z.L.)

**Keywords:** *Zanthoxylum bungeanum*, Tibetan pig, protein structure, texture, polyphenol, Chinese sausages, protein intermolecular force

## Abstract

The mechanistic effects of *Zanthoxylum bungeanum* on the textural properties of Tibetan pig sausages remain inadequately elucidated. We conducted a dose–response analysis using *Z. bungeanum* aqueous extraction (ZBAE) containing 36.97 mg GAE/g polyphenols, applied at concentrations from 0.125% to 1.20% in the meat paste. Optimal textural enhancement was achieved at 0.25% ZBAE, as proved by significantly improving water holding capacity (1.77% increase), hardness (139.87% increase), and gel strength (46.04% increase) relative to the control group (*p* < 0.05). Specifically, this concentration: (i) promoted protein molecular rearrangement of by enhancing hydrophobic interactions (33.33% increase) and hydrogen bonding (287.99% increase); (ii) induced conformational transitions from *α*-helix (42.62% decrease) to *β*-sheet formations (21.11% increase); and (iii) generated a homogeneous three-dimensional protein network characterized by a fractal dimension of 2.769 ± 0.006 and a porosity of 38.350 ± 0.333%. The addition of natural polyphenols from Z. *bungeanum* may optimize the textural quality of processed meat products.

## 1. Introduction

Chinese sausages, a highly esteemed traditional meat product, have gained widespread popularity across Asia [1]. Among these, Sichuan sausage, characterized by the incorporation of *Zanthoxylum bungeanum* (commonly known as Sichuan pepper or *huajiao*), stands out as the most prominent traditional processed meat food in southwestern China [2]. *Z. bungeanum* has been frequently utilized as a flavoring agent in traditional Chinese cuisine [3]. The distinctive sensory attributes, particularly the numbing sensation and aroma, have driven its growing popularity and acceptance within the Chinese modern food industry.

*Z. bungeanum* has exhibited promising applications in meat processing, particularly in enhancing the structure and modulating the functionality of meat proteins [4]. The various natural compounds present in *Z. bungeanum*, primarily polyphenols, alkaloids, and polysaccharides, are reported to interact with the myofibrillar proteins of meat [5]. These interactions potentially alter the protein conformational stability, cross-linking configurations, and solubility [6]. *Β*-sanshool in *Z. bungeanum* modifies myofibrillar proteins, thereby influencing the quality characteristics of processed meat [7]. The structure–activity relationship and the underlying mechanism of affinity between flavonols in *Z. bungeanum* and myofibrillar proteins have been examined [8]. Additionally, *Z. bungeanum* essential oil demonstrates potential in reducing cooking losses, enhancing water-holding capacity, and inhibiting protein degradation [9].

Presently, systematic investigation into the quality attributes of Tibetan pig remains limited [10]. As the only swine breed adapted to high-altitude grazing on the Qinghai–Tibet Plateau [11], Tibetan pigs have evolved under a cold, arid climate at elevations ranging from 2500 to 4100 m [12]. Tibetan pork is highly esteemed by consumers in southwestern China for its superior flavor and quality, including its enhanced water retention, superior red coloration, abundant flavor amino acids, elevated inosine monophosphate content, smaller muscle fiber area, and higher density. These superior quality attributes are hypothesized to result from the adaptive glycolysis metabolism rates [13].

Proteins serve as the fundamental structural components and undergo a series of physicochemical transformations during sausage production, significantly influencing the final texture and functionality [14]. Polyphenols, characterized by their aromatic rings and hydroxyl groups, demonstrate strong reactivity with proteins. Upon interaction, the surface characteristics, conformation, and supramolecular architecture of proteins are modified by polyphenols [15], leading to the formation of a distinct textural quality. Studies suggest that polyphenols may alter the secondary and tertiary structures of proteins, thereby enhancing their functional properties by strengthening water–protein and protein–protein interactions [16]. However, systematic investigations examining the impact of *Z. bungeanum* polyphenols on the textural attributes of pork sausages remain limited.

Therefore, this study evaluated the incorporation of various concentrations of *Z. bungeanum* aqueous extract (ZBAE) into Tibetan pig sausages. Comprehensive analyses were conducted on the alterations in water holding capacity, color, texture, gel strength, intermolecular forces, moisture distribution patterns, protein secondary structures, and microstructure. This investigation aims to assess the impact of ZBAE on sausage textural properties and elucidate the underlying mechanisms through which polyphenols in *Z. bungeanum* modify pork protein structure. This work may provide novel insights into the multifunctional potential of *Z. bungeanum* as a natural additive in meat preservation and product innovation.

## 2. Materials and Methods

### 2.1. Materials and Reagents

*Zanthoxylum bungeanum* was procured from Yonghui supermarket (Hefei, China). Hind leg meat and backfat were randomly sampled from six Tibetan pigs (average weight of 50 ± 2 kg) in Shannan Ruju Agricultural Technology Co., Ltd. (Tibet, China), vacuum-packed, and stored at −18 ± 2 °C. PAPE casings with a fold diameter of 40 mm and an inner diameter of 25 mm were obtained from Laoling Yingyuanxiang Food Co., Ltd. (Dezhou, China).

Sodium dodecyl sulfate and *β*-mercaptoethanol were obtained from Sinopharm Chemical Reagent Co., Ltd. (Shanghai, China); tris(hydroxymethyl)aminomethane, glycine, and bromophenol blue were obtained from Biosharp Co. (Hefei, China); Bradford kit was obtained from Meilun Biological Technology Co., Ltd. (Dalian, China); protein spectral marker was obtained from Solarbio Co. (Beijing, China). All other chemicals and reagents were of analytical grade and obtained from Aladdin Biochemical Technology Co., Ltd. (Shanghai, China).

### 2.2. Preparation of Zanthoxylum bungeanum Aqueous Extract

*Zanthoxylum bungeanum* aqueous extract (ZBAE) was prepared according to the method of Zhu, Liu, He, Wang, and Li [4] with modifications. The seeds were oven-dried at 70 °C for 48 h and then cooled to room temperature. They were pulverized to a 50-mesh particle size and mixed with deionized water at a ratio of 1:10 (*w*/*v*). Ultrasonic extraction was carried out at 50 °C, 200 W for 90 min. After filtration, the filtrate was concentrated using an Eyela N-1300 rotary evaporator (Rikakikai, Co., Ltd., Tokyo, Japan) at 45 °C with a rotation speed of 90 r/min. The concentrate was lyophilized into powder in a PD-ICE equipment (Detianyou Technology Development Co., Ltd., Beijing, China), subsequently stored at −20 °C.

### 2.3. Determination of Total Phenolic Content in ZBAE

The total phenolic content of ZBAE was quantified using the Folin–Ciocalteu method [17] with modifications. Briefly, 0.1 mL of the extract was mixed with 2 mL of 20% Na_2_CO_3_ solution and incubated at 25 ± 1 °C for 2 min. Subsequently, 0.9 mL of Folin–Ciocalteu reagent was added and thoroughly mixed. The absorbance at 765 nm was measured after incubation of 30 min. The total phenolic content was expressed as milligrams of gallic acid equivalents per gram of sample (mg GAE/g), using a standard curve prepared with gallic acid.

### 2.4. Preparation of Tibetan Pig Sausage with ZBAE

Lean meat and fat were mixed at a ratio of 95:5 after removing the connective tissue. NaCl was added to the mixture at a concentration of 6% (*w*/*w*). The mixture was then ground into meat paste at 4 °C. ZBAE powder was dissolved in deionized water, and added into the paste to achieve final concentrations of 0.125%, 0.25%, 0.50%, 1.00%, and 1.20% (*w*/*w*), respectively. A control group without ZBAE was also prepared. Each treatment and control group consisted of 2.0 kg of meat paste. After blending, the paste was stuffed into PAPE casings with a fold diameter of 40 mm and an inner diameter of 25 mm. The sausages were equilibrated at 4 °C for 4 h, then dried and aged in a DHG-9245A electric drying oven (Yiheng Scientific Instrument Co., Ltd., Shanghai, China) at 45 °C for 3 h, with hourly turning. Finally, the sausages were vacuum-packed and stored at 0~4 °C.

### 2.5. Water Holding Capacity

The water holding capacity (WHC) was evaluated by determining the water retention of the gel after centrifugation. Approximately 5 g of the sample was wrapped in three layers of filter paper and centrifuged at 6000× *g* for 15 min at 4 °C using a H1750R centrifuge (Xiangyi Laboratory Instrument Development Co., Ltd., Changsha, China). The mass of the sample was recorded both before and after centrifugation. WHC was calculated as the percentage of mass lost after centrifugation, relative to the original mass [18].

### 2.6. Color

Surface color values were measured using a CR-400 Chromameter (Konica-Minolta Inc., Tokyo, Japan) following the method of Bae and Jeong [19] with modifications. Before measurements, the instrument was calibrated against a standard white plate. Colorimetric analysis was performed under illuminant D_65_, a 2° standard observer, and an 8 mm aperture size. The recorded parameters included L* (brightness), a* (red/green), and b* (yellow/blue).

### 2.7. Texture Profile Analysis

Textural properties were analyzed using a TA-XT Plus texture analyzer (Stable Micro Systems, Godalming, UK) equipped with a cylindrical probe (P/36R), following the method of Zhou, et al. [20]. Sample was equilibrated at 25 ± 1 °C for 1 h prior to testing, then cut into cylinders with a height and diameter of 3.5 cm. Texture profile analysis was performed under the following parameters: trigger force of 10.0 g; pre-test speed of 1.0 mm/s; test speed of 5.0 mm/s; post-test speed of 5.0 mm/s; and compression ratio was set to 20%.

### 2.8. Puncture Test

Gel strength was quantified using a puncture test on the same TA-XT Plus texture analyzer (Stable Micro Systems, Godalming, UK) equipped with a P/5 s spherical plunger probe, following the method of Yi, et al. [21]. The sample was cut into cylinders with a height and diameter of 25 mm, and equilibrated at 25 ± 1 °C for 1 h. Analysis was performed under the following parameters: trigger force of 5.0 g, pre-test speed of 2.0 mm/s, test speed of 1.0 mm/s, return speed of 10.0 mm/s, and deformation of 45%. The maximum force recorded during probe penetration was defined as gel strength.

### 2.9. Intermolecular Forces

Non-covalent interactions, disulfide bond, and sulfhydryl group contents in sausage gels were quantified according to the modified method of Yan, et al. [22], with modifications.

Non-covalent interactions were analyzed using five solutions: 0.05 mol/L NaCl (SA), 0.6 mol/L NaCl (SB), 1.5 mol/L urea solution + 0.6 mol/L NaCl (SC), 8 mol/L urea solution + 0.6 mol/L NaCl (SD), and 8 mol/L urea solution + 0.6 mol/L NaCl + 0.5 mol/L 2-*β*-mercaptoethanol (SE). Subsequently, 2 g of sample was minced and defatted by washing with ethanol and diethyl ether twice, respectively. After solvent evaporation, the sample was homogenized in 10 mL of each solution at 4 °C for 3 min. After 1 h stirring at 4 °C, suspension was centrifuged at 6000× *g* for 15 min. Protein content in the supernatant was measured via Bradford assay. The differences in protein content among the supernatants indicated the presence of various non-covalent interactions: ionic bonds (difference between SB and SA), hydrogen bonds (difference between SC and SB), and hydrophobic interactions (difference between SD and SC). The disulfide bond content was determined by the difference between SE and SD.

For the determination of sulfhydryl groups, 0.5 mL of the SD supernatant (at a concentration of 4 mg/mL) was added to 4.5 mL of 0.2 M Tris-HCl buffer (pH 8.0) containing 8 M urea, 5 mM EDTA, and 1% SDS. After thoroughly mixing, 4 mL of this mixture was combined with 0.4 mL of a solution containing 10 mM DTNB and 10 mM Tris-HCl (pH 8.0), and incubated at 40 °C for 30 min. The absorbance at 412 nm was measured using a UV-5500 spectrophotometer (Metash Instrument Co., Ltd., Shanghai, China) with a 0.6 M KCl solution serving as the blank. The content of the sulfhydryl group was calculated as:

Sulfhydryl group content (mol/10^5^ g protein) = 73.53 × A_412_ × D/*c*

where 73.53 is derived from 10^6^/13,600, with 13,600 being the molar extinction coefficient of Ellman’s reagent; A_412_ represents the absorbance measured at 412 nm; D is the dilution factor; *c* is the protein concentration.

### 2.10. Moisture Distribution

Moisture distribution was analyzed using a MesoMR23-060H-I low-field nuclear magnetic resonance (LF-NMR) instrument (Suzhou Newmark Analytical Instruments Co., Suzhou, China) with the modified method of Meng, et al. [23]. Before measurement, samples were equilibrated at 25 ± 1 °C for 30 min and then cut into cylinders (diameter of 10 mm; height of 20 mm). These cylinders were loaded into NMR tubes and subjected to a Carr–Purcell–Meiboom–Gill pulse sequence with the following parameters: resonance frequency of 23 MHz; wait time of 1800 ms; echo time of 0.2 ms; 8 scans; and 4000 echoes collected.

### 2.11. Sodium Dodecyl Sulfate Polyacrylamide Gel Electrophoresis (SDS-PAGE)

SDS-PAGE was conducted according to the method by Zhou, Yang, Yin, Huang, Yan, Zhang, and Xie [20], using a vertical slab electrophoresis unit (Junyidongfang, Beijing, China). The gel matrix consisted of 15% separating gel and 5% stacking gel. Electrophoresis separation was performed under a constant voltage of 200 V for 150 min. After electrophoresis, protein was visualized through staining with a solution containing 0.1% Coomassie Brilliant Blue R-250, 25% isopropanol, and 15% acetic acid for 30 min. Subsequently, the gels were destained with a solution containing 5% anhydrous alcohol and 10% acetic acid for 1 h. The separated protein bands were identified by comparing them with a standard protein marker, which had a molecular weight range of 11~245 kDa (Solebold Technology Co., Ltd., Beijing, China).

### 2.12. Protein Secondary Structure

The protein secondary structure of the samples was analyzed using a Nicolet iS20 Fourier transform infrared spectrometer (Thermo Fisher Scientific, MA, USA) with the modified method by Zhao, et al. [24]. Lyophilized sample powder was blended with KBr at a mass ratio of 1:100. The mixture was then pressed under a pressure of 29 MPa for 1 min. The measurement was conducted under the following parameters: a resolution of 4 cm^−1^, 32 scans, and a spectral range of 4000~500 cm^−1^. To qualify the protein secondary structure, the amide I band was deconvoluted using PeakFit software version v4.12 (Seasolve, Framingham, MA, USA). Second derivative spectra and Gaussian fitting were performed in the range of 1695~1660 cm^−1^ to obtain detailed information about the secondary structure components.

### 2.13. Scanning Electron Microscopy

Microstructural characterization of sausage samples was conducted using the method of Zhou, Yang, Yin, Huang, Yan, Zhang, and Xie [20] on a Hitachi SU8010 field emission scanning electron microscope (Carl Zeiss AG Aktien Gesellschaft, Jena, Germany). Cubic samples (5 × 5 × 2 mm) were prepared and immersed in 2.5% (*v*/*v*) glutaraldehyde solution (pH 6.8) at 4 °C for 24 h. Subsequently, the samples were rinsed three times with 0.1 mol/L phosphate buffer (pH 7.0) for 10 min each, then dehydrated in a series of graded ethanol concentrations (30%, 50%, 70%, 80%, 90%, and 100%) for 15 min each. Following dehydration, the samples were subjected to a K850 critical point dryer (Quorum Technologies, East Sussex, UK), then mounted and coated with gold. The fractal dimension (Df) of the SEM images was measured using ImageJ software (version v1.5.2a, National Institutes of Health and the Laboratory for Optical and Computational Instrumentation, Madison, WI, USA) with its Fractal Box Count plug-in. Before analysis, the SEM images were thresholded and binarized. Df and porosity were calculated according to the method of Liu, et al. [25] as follows:

D = −lg Nε/lgε

Df = D + 1

where Nε represents the number of boxes containing target pixels at a specific scale; D represents the slope of the line; and ε represents the corresponding scale.

### 2.14. Statistical Analysis

At least three independent measurements were performed using sausages from different batches to ensure the reliability of the results. Data are expressed as the mean ± SD. Statistical significance was assessed by one-way ANOVA, followed by Duncan’s multiple range tests at the 5% level of significance, using SPSS version 22.0 (IBM Co., New York, NY, USA).

## 3. Results and Discussion

### 3.1. Effects of Different ZBAE Concentrations on the Physicochemical Attributes of Tibetan Pig Sausages

Table 1 illustrates the comprehensive evaluation of Tibetan pig sausage physicochemical attributes, including water holding capacity (WHC), color values, textural properties, and puncture test, across ZBAE concentrations ranging from 0% to 1.20%. The total phenolic content of ZBAE powder was determined to be 36.97 ± 1.45 mg GAE/g, which aligns with the previous report of dried *Zanthoxylum bungeanum* Maxim. [26].

#### 3.1.1. WHC

WHC reflects the protein’s capacity to retain moisture, which is a crucial indicator of meat product texture and juiciness [18]. As shown in Table 1, the WHC initially increased and then decreased with rising ZBAE concentration (0%~1.20%), which generally aligns with the effects of polyphenols in lotus root knot extract on fish protein gel [27]. Sample with 0.25% ZBAE showed the highest WHC of 1.77% improvement compared with the control group, likely due to the antioxidant protection against protein oxidation-induced hydrophilicity loss during drying and aging [28]. In comparison to the control group, 1.00% ZBAE did not alter WHC (*p* > 0.05), while 1.20% ZBAE significantly reduced WHC (*p* < 0.05), potentially indicating destabilization of the protein network when high concentration of extracts or phenolic compounds was added [29].

#### 3.1.2. Color

Color is a crucial aspect of the sensory properties that significantly influences consumer preferences for meat products [30]. The brightness (L*) value gradually decreased from 42.27 ± 0.29 to the minimum of 37.75 ± 1.11 when the ZBAE concentration rose to 1.2%, and this trend was similar to the report of Zhu, Liu, He, Wang, and Li [4]. This decline may be attributed to moisture loss, nitrite fermentation, or altered light scattering on the cut surface of sausages [31,32]. The added ZBAE did not affect the redness (a*) value (*p* > 0.05), except for the concentration of 1.2% (*p* < 0.05). Typically, the a* value is influenced by the concentration and redox state of heme pigment and myoglobin, especially the content of nitroso myoglobin [33]. Concurrently, the yellowness (b*) value rose and peaked at a ZBAE concentration of 1.00%. This observation aligns with the increase in the b* value of beef jerky with *Z. bungeanum* extract addition [4]. Several factors might contribute to this increase, such as the inherent light red-brown shade of ZBAE, the Maillard reaction that occurred during drying, or the oxidation of polyphenols and other organic components in ZBAE [4].

#### 3.1.3. Textural Attributes Analysis

Compared to the control group, ZBAE significantly increased all tested parameters (*p* < 0.05), except for cohesiveness in the 0.125% group and resilience in the 0.125%, 1.00%, and 1.20% groups. As the concentration of ZBAE increased, the textural parameters mostly exhibited an initial increase followed by a decrease. At ZBAE concentrations of 0.25% and 0.50%, polyphenols appeared to positively influence sausage texture, which might facilitate the formation of a dense and homogeneous network structure through cross-linking. This structure improved the ability to retain non-flowing water, decreased the movement of free water [34,35], and consequently enhanced the WHC (Section 3.1.1). Hardness significantly increased after the addition of ZBAE and peaked at a concentration of 0.25% across all samples (*p* < 0.05). This aligns with the increase in hardness observed in pork sausages caused by phenolic compounds [36]. Specifically, the addition of 0.25% ZBAE enhanced the hardness of Tibetan pig sausage by 139.87%, compared to the control group. Springiness, which indicates the physical resilience after initial compression, reached its maximum value at 0.50% concentration (*p* < 0.05), alongside cohesiveness and resilience. Moreover, both gumminess and chewiness attained the highest levels at 0.25% and 0.50% ZBAE (*p* < 0.05). This enhancement of texture is likely due to the formation of stable bonds between the polyphenols in ZBAE and proteins, fats, and water through various interactions [37].

#### 3.1.4. Puncture Test

Puncture test is a reliable method for evaluating the gel strength of various sausages [18]. Gel strength initially increased and then decreased with rising ZBAE additions, which is similar to the WHC trend observed in Section 3.1.1. And the maximum value was achieved at a ZBAE concentration of 0.25%, where the polyphenols improved the gel strength of Tibetan pig sausage by 46.04%, relative to the control group (*p* < 0.05). Generally, elevated gel strength reflects acceptable protein structure within a certain range. However, excessive gel strength does not necessarily equate to superior gel quality. An overly hard gel may result from low moisture content and an uneven internal structure [18].

### 3.2. Effects of Different ZBAE Concentrations on the Intermolecular Forces of Tibetan Pig Sausages

#### 3.2.1. Non-Covalent Interaction Forces

**Figure 1:**
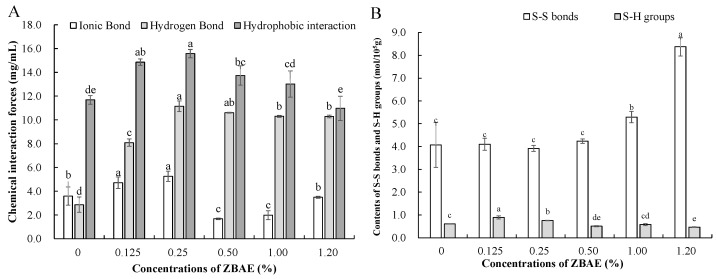
Chemical interaction forces (**A**), sulfhydryl groups and disulfide bonds contents (**B**) of sausage samples. ZBAE: *Zanthoxylum bungeanum* aqueous extract. Different letters above the columns indicate that they are significantly different by Duncan’s multiple range tests (*p* < 0.05).

Figure 1A illustrates the effects of ZBAE at selected concentrations on non-covalent interaction forces. Although the effects differed depending on the additive concentrations, most forces were enhanced compared to the control group. Notably, all three forces peaked at a ZBAE concentration of 0.25%, corresponding with the optimal WHC and gel strength values (Table 1), consistent with previous reports [30]. Compared to the control group, hydrophobic interactions increased by 33.33%, and hydrogen bonding exhibited a more pronounced increase of 287.99%. Phenol-induced non-covalent interactions, particularly those mediated by phenolic hydroxyl groups, significantly contribute to gel stabilization and water retention enhancement. The order of non-covalent forces in the treated samples was as follows: hydrophobic interactions > hydrogen bonds > ionic bonds. This suggests that hydrophobic interactions play a dominant role in maintaining the stability of protein–polyphenol conjugates and the structural integrity of the gel texture [38]. Hydrogen bonds were significantly elevated across all ZBAE-treated samples (*p* < 0.05), primarily attributed to interactions between phenolic hydroxyl groups in ZBAE and hydrogen acceptors in proteins [39]. In contrast, ionic bonds exhibited no clear concentration-dependent trend, likely due to their susceptibility to thermal disruption and potential to alter the local structural reorientation of protein residues [37].

#### 3.2.2. Sulfhydryl Group (SH) and Disulfide Bonds Contents

Free SH groups are crucial determinants of protein functionality, affecting water retention, gel texture, and antioxidant stability [5]. As shown in Figure 1B, SH content was significantly elevated at ZBAE concentrations of 0.125% and 0.25% relative to the control group (*p* < 0.05), suggesting that polyphenols may inhibit SH oxidation through scavenging free radicals [40]. This aligns with previous studies on rutin and quercetin protecting SH groups in porcine myofibrillar proteins [41]. Sulfhydryl groups, particularly those with accessible free thiol groups on cysteine residues, are intimately correlated with protein oxidation [4,40]. However, at ZBAE concentrations exceeding 0.25%, SH group content decreased significantly compared to the control group (*p* < 0.05). This may be attributed to the auto-oxidation of excess polyphenols at high concentrations, generating reactive quinones that covalently bind SH groups into mercaptoquinone adducts [42]. The concurrent increased disulfide bond contents observed in the 1.00% and 1.20% ZBAE groups (Figure 1B) are likely due to structural unfolding, exposing SH groups and facilitating their oxidation into intra- or intermolecular disulfide bonds [43]. The excessive disulfide bonding, however, compromised gel network integrity by inducing over-cross-linking and brittleness [44]. The inverse correlation between SH groups and disulfide bonds relatively supported the redox-driven structural alterations observed in Figure 1B.

### 3.3. Effects of Different ZBAE Concentrations on the Water State of Tibetan Pig Sausages

**Figure 2:**
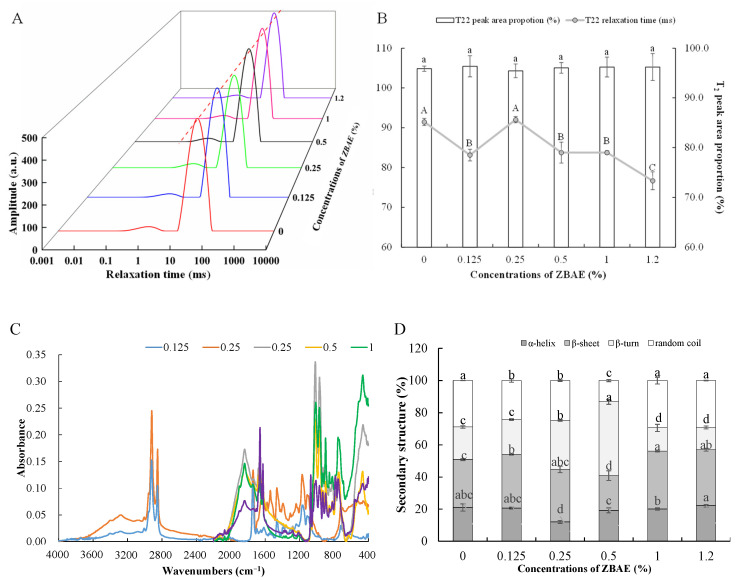
Distribution of T_22_ relaxation times (**A**), relaxation peak area proportions (**B**), infrared spectra (**C**), and secondary structure composition (**D**) in sausage samples. ZBAE: *Zanthoxylum bungeanum* aqueous extract. Different letters indicate significant differences among groups of different ZBAE contents by Duncan’s multiple range tests (*p* < 0.05).

Low-field nuclear magnetic resonance (LF-NMR) was used to analyze the mobility and distribution of water molecules in the sausage matrix, indicating their interactions with exchangeable protein-bound protons. The state of water significantly influences sausage quality properties, including hardness and surface texture [37]. Figure 2A illustrates two peaks within the 0~10,000 ms range, corresponding to bound water (T_21_, 0.01~10 ms) and immobilized water (T_22_, 40~300 ms). The consistent two peaks across all samples suggest that the ZBAE addition does not modify the water mobility pattern. Free water was absent across all treatments, probably because of dehydrating during the drying process. Bound water (T_21_) is strongly correlated with macromolecules such as proteins and polysaccharides, while immobilized water (T_22_) is confined within the gel network, exhibiting restricted mobility [45]. A leftward shift in T_22_ relaxation times was observed across all treatments except for the 0.25% ZBAE group, suggesting reduced free hydrogen protons, and strengthened protein–water interactions [46]. Figure 2B illustrates the relative proportions of different water forms. The addition of ZBAE at varying concentrations exhibited a negligible effect on T_22_ peak area proportions, which consistently exceeded 95% across all samples, emphasizing the dominance of immobilized water in the sausage matrix.

### 3.4. Effects of Different ZBAE Concentrations on the Protein Structure of Tibetan Pig Sausages

#### 3.4.1. Sodium Dodecyl Sulfate Polyacrylamide Gel Electrophoresis (SDS-PAGE)

**Figure 3:**
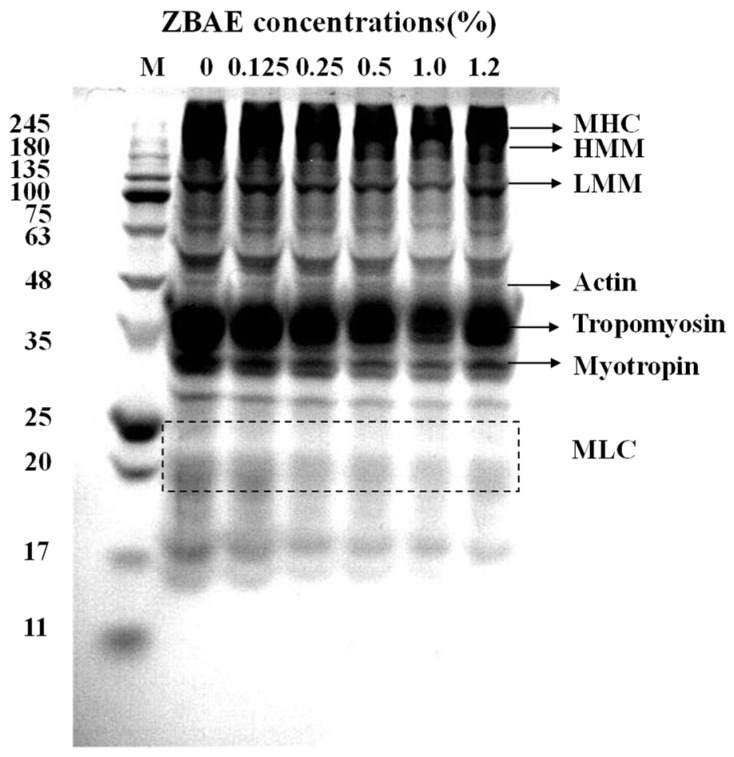
Protein profile of sausage samples through SDS-PAGE. ZBAE: *Zanthoxylum bungeanum* aqueous extract; M: marker; MHC: myosin heavy chain; HMM: heavy meromyosin; LMM: light meromyosin; MLC: myosin light chain.

SDS-PAGE (Figure 3) was performed to elucidate the protein profiles and subunit composition in ZBAE-supplemented sausages. Electrophoretic patterns revealed five major protein bands: myosin heavy chain (~240 kDa), heavy meromyosin (170 kDa), tropomyosin (35 kDa), light meromyosin (~100 kDa), and trace amounts of myosin light chain. Different ZBAE concentrations barely affected the intensity distribution across all tested samples. At 1.00% ZBAE concentration, bands corresponding to light meromyosin, myotropin, and myosin light chains slightly diminished, likely due to polyphenol-induced protein cross-linking, which obscures electrophoretic migration. It is possible that minor cross-linking between organic compounds in ZBAE and myosin occurs during sausage gelation and drying [30], leading to the dissociation of light meromyosin, myotropin, and myosin light chains. Furthermore, ZBAE compounds might promote protein polymerization, resulting in the formation of high molecular weight aggregates that are too large to penetrate the stacking gel [47], thereby leading to a reduction in visible protein bands. The intensities of major bands slightly increased at 1.20% ZBAE concentration, which may be attributed to enhanced myosin aggregation correlated with increased disulfide bond formation during the gelation of pork (Figure 1B). Furthermore, ZBAE at higher concentrations may decelerate the degradation of myofibrillar proteins by inhibiting microbial enzyme activity [4].

#### 3.4.2. Protein Secondary Structure

Fourier transform infrared spectroscopy (FTIR) analysis can be utilized to assess alterations in the secondary structures of proteins [48]. The spectra include the amide A region (3300 cm^−1^, corresponding to N-H stretching vibrations), the amide B region (2961 cm^−1^, corresponding to C-N stretching vibrations), the amide I region (1700~1600 cm^−1^, corresponding to C=O stretching vibrations), the amide II region (1600~1500 cm^−1^, corresponding to N-H bending vibrations), and the amide III region (1360~1200 cm^−1^, corresponding to C-N and N-H stretching vibrations) [49]. Specifically, the spectral ranges of 1651~1660 cm^−1^, 1600~1640 cm^−1^, 1661~1700 cm^−1^, and 1641~1650 cm^−1^ represent *α*-helix, *β*-sheet, *β*-turn, and random coil structures, respectively [50].

As illustrated in Figure 2C, the absorption peaks of sausage samples varied significantly across different ZBAE concentrations. The effects of ZBAE on the relative percentages of secondary structures are depicted in Figure 2D, which varied depending on the additional amount of ZBAE. The relative content of *α*-helix reached its minimum of 12.08 ± 0.93% (a decrease of 42.62% compared to the control) at a concentration of 0.25%, transforming into relatively disordered *β*-sheet (an increase of 21.11% compared to the control) and *β*-turn structures (*p* < 0.05). Similarly, 0.50% ZBAE addition also led to a decrease in *α*-helix content compared to the control. However, the formation of random coils was potentially inhibited at 0.125%, 0.25%, and 0.50% ZBAE additions, maintaining more stable and organized network structures. The concentration of the *α*-helix structure exhibited an inverse correlation with gel strength in Table 1, similar to the report of Mi, et al. [51]. The hydroxyl groups in polyphenols might interfere with the *α*-helix structure, which is primarily stabilized by hydrogen bonds between the carbonyl oxygen and amino hydrogens of amino acids [52]. The unfolding and untwisting of the *α*-helix structure exposed more internal groups, potentially enhancing intermolecular interactions.

Furthermore, the *β*-sheet content exhibited a statistical increase across all treatments (*p* < 0.05), except for the group of 0.50% ZBAE. It is hypothesized that the hydroxyl groups produced through oxidation might interfere with hydrogen bonds, thereby facilitating the unwinding of both *α*-helix and *β*-sheet structures [53]. Regarding the *β*-turn content, it increased at both 0.25% and 0.50% addition levels. The random coil configuration predominantly decreased in the 0.50% ZBAE group. This observation basically aligns with previous studies on the effects of pepper leaf polyphenol additions [5].

#### 3.4.3. Scanning Electron Microscope

**Figure 4:**
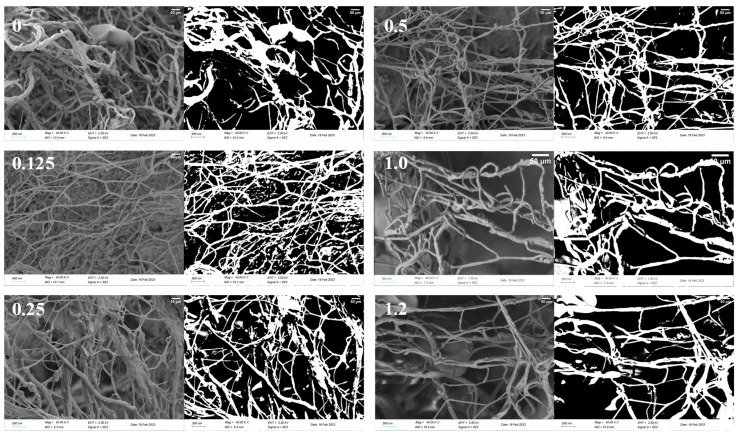
Cross-sectional electron microscope images and binary images of sausages at different *Zanthoxylum bungeanum* aqueous extract concentrations (%).

The interactive and homogeneous microstructure of proteins plays a crucial role in WHC and other characteristics, serving as an indicator of gel properties [54]. Figure 4 presents cross-sectional electron micrographs of the gel network in Tibetan pig sausages with different concentrations of ZBAE, and their corresponding binary images. In these images, the white areas indicate the myofibrillar protein gel network, while the black areas indicate the pores within the network. The control group exhibited a rough structure with unevenly distributed large pores, through which water and fat are lost during protein denaturation in the drying process. Conversely, the incorporation of 0.125% and 0.25% ZBAE resulted in a more defined gel network compared to the control group, thereby improving the microstructure. The fractal dimension (Df) value presented in Table 2 reflects the extent of protein aggregation, which may potentially influence macroscopic properties. Samples containing 0.125% ZBAE concentrations exhibited the highest Df values across all samples (*p* < 0.05), indicating robust cross-linking and a dense, uniform pore size distribution within a scattered network structure. Typically, a more refined microstructure is positively correlated with WHC and gel strength [27], as shown in Table 1. The polyphenols in ZBAE possibly form covalent bonds with proteins, thereby enhancing cross-linking between protein molecules. This contributes to smaller pore sizes on the gel surface and organized dense network structure, similar to the reduction in surface roughness and unevenness observed in betel leaf extract-treated buffalo sausages [55]. However, porosity gradually decreased when the ZBAE concentration exceeded 0.50%, until the lowest levels at the concentration of 1.2% across all samples (*p* < 0.05), indicating that the pores became uneven and larger, and the surface appeared rough. Notably, the 0.25% ZBAE treatment group developed a homogeneous three-dimensional network with a fractal dimension of 2.769 ± 0.006 and a porosity of 38.350 ± 0.333%. This phenomenon is also depicted in Figure 4, consistent with the observed decreases in WHC, gel strength, and textural parameters such as hardness, as discussed in Section 3.1.

In treatments where ZBAE concentrations exceed 1.00%, the excessive interaction between polyphenols and proteins may lead to rapid and disorganized aggregations. This could impede protein cross-linking, thereby disrupting the protein network structure and making it loose, ultimately degrading the textural properties [56].

## 4. Conclusions

The incorporation of an optimal concentration of *Zanthoxylum bungeanum* aqueous extract (ZBAE) into Tibetan pig sausage significantly enhances its water holding capacity (WHC), gel strength, and textural properties, specifically the concentration at 0.25%. This enhancement is attributed to ZBAE’s ability to foster hydrophobic interactions and protect active sulfhydryl groups from converting into disulfide bonds, potentially by inhibiting protein aggregation through retardation of amino acid residue oxidation. Furthermore, the addition of 0.25% ZBAE contributed to the development of a dense pore structure with a smooth surface in the sausage gel. However, excessive addition of ZBAE impeded protein binding, leading to decreased gel strength. This study primarily focused on examining the physicochemical and sensory properties of Tibetan pig sausages supplemented with polyphenols from ZBAE. However, further investigation using a pure protein system is necessary to simplify the matrix and gain a more comprehensive understanding of the improvement in Tibetan pig products.

## Figures and Tables

**Table 1:** Water holding capacity, color, texture, and gel strength of sausage samples.

Concentrations of ZBAE (%)	0	0.125	0.25	0.50	1.00	1.20
Water holding capacity (%)	94.40 ± 0.16 ^d^	94.87 ± 0.09 ^c^	96.07 ± 0.09 ^a^	95.13 ± 0.09 ^b^	94.53 ± 0.09 ^d^	94.00 ± 0.16 ^e^
L*	42.27 ± 0.29 ^a^	41.01 ± 0.68 ^abc^	41.50 ± 0.17 ^b^	40.46 ± 0.54 ^cd^	39.61 ± 0.43 ^de^	37.75 ± 1.11 ^e^
a*	11.20 ± 0.25 ^a^	11.17 ± 0.08 ^a^	11.41 ± 0.43 ^a^	10.53 ± 0.56 ^ab^	9.72 ± 0.96 ^ab^	9.07 ± 1.04 ^b^
b*	9.42 ± 0.49 ^c^	10.11 ± 0.32 ^bc^	10.24 ± 0.23 ^b^	10.07 ± 0.61 ^bc^	11.31 ± 0.41 ^a^	10.10 ± 0.74 ^bc^
Hardness (g)	3449.94 ± 25.70 ^e^	4480.61 ± 15.44 ^c^	8275.25 ± 19.13 ^a^	7400.55 ± 3.88 ^b^	4501.26 ± 3.58 ^c^	4270.16 ± 31.97 ^d^
Springiness (%)	0.561 ± 0.008 ^d^	0.543 ± 0.025 ^d^	0.689 ± 0.004 ^b^	0.771 ± 0.005 ^a^	0.667 ± 0.004 ^bc^	0.655 ± 0.005 ^c^
Cohesiveness	0.427 ± 0.005 ^d^	0.382 ± 0.007 ^e^	0.476 ± 0.002 ^b^	0.536 ± 0.002 ^a^	0.445 ± 0.002 ^c^	0.434 ± 0.005 ^d^
Gumminess	1452.80 ± 21.27 ^e^	1788.61 ± 48.37 ^d^	3894.61 ± 59.06 ^a^	3957.68 ± 24.99 ^a^	1976.94 ± 26.81 ^c^	2067.92 ± 19.44 ^b^
Chewiness	780.70 ± 3.85 ^d^	885.10 ± 11.83 ^c^	3054.23 ± 18.95 ^a^	3081.40 ± 13.57 ^a^	1359.00 ± 5.92 ^b^	1359.15 ± 27.06 ^b^
Resilience (%)	0.138 ± 0.001 ^c^	0.093 ± 0.002 ^e^	0.148 ± 0.002 ^b^	0.170 ± 0.002 ^a^	0.118 ± 0.001 ^d^	0.116 ± 0.004 ^d^
Gel strength (g·cm)	1101.01 ± 5.66 ^c^	1106.04 ± 1.84 ^c^	1607.87 ± 9.73 ^a^	1180.42 ± 9.36 ^b^	1071.28 ± 8.48 ^d^	1056.02 ± 9.84 ^d^

ZBAE: *Zanthoxylum bungeanum* aqueous extract. The results are expressed as mean ± standard deviation. Different letters in the same row indicate that the properties are significantly different by Duncan’s multiple range tests (*p* < 0.05).

**Table 2:** Df and porosity of sausage samples.

Concentrations of ZBAE (%)	0	0.125	0.25	0.50	1.00	1.20
Fraction dimension	2.748 ± 0.003 ^cd^	2.814 ± 0.001 ^a^	2.769 ± 0.006 ^b^	2.757 ± 0.000 ^c^	2.749 ± 0.005 ^cd^	2.747 ± 0.002 ^d^
Porosity (%)	37.597 ± 0.438 ^b^	38.48 ± 0.090 ^a^	38.350 ± 0.333 ^a^	37.670 ± 0.204 ^b^	35.873 ± 0.109 ^c^	34.350 ± 0.151 ^d^

Df: fraction dimension; ZBAE: *Zanthoxylum bungeanum* aqueous extract. The results are expressed as mean ± standard deviation. Different letters in the same row indicate that the properties are significantly different by Duncan’s multiple range tests (*p* < 0.05).

## Data Availability

The original contributions presented in this study are included in the article. Further inquiries can be directed to the corresponding author.

## Abbreviations

The following abbreviations are used in this manuscript:

ZBAE	*Zanthoxylum bungeanum* aqueous extract
WHC	Water holding capacity
SH	Sulfhydryl
FTIR	Fourier transform infrared spectra
SDS-PAGE	Sodium dodecyl sulfate polyacrylamide gel electrophoresis
LF-NMR	Low-field nuclear magnetic resonance
MHC	Myosin heavy chain
MLC	Myosin light chain
Df	Fraction dimension

## References

[1] Xiao Y., Liu Y., Chen C., Xie T., Li P. (2020). Effect of Lactobacillus plantarum and Staphylococcus xylosus on flavour development and bacterial communities in Chinese dry fermented sausages. Food Res. Int..

[2] Chen X., Yan F., Qu D., Wan T., Xi L., Hu C.Y. (2024). Aroma characterization of Sichuan and Cantonese sausages using electronic nose, gas chromatography–mass spectrometry, gas chromatography-olfactometry, odor activity values and metagenomic. Food Chem. X.

[3] Jia W., Wang X. (2023). *Zanthoxylum bungeanum* as a natural pickling spice alleviates health risks in animal-derived foods via up-regulating glutathione S-transferase, down-regulating cytochrome P450 1A. Food Chem..

[4] Zhu H., Liu F., He L., Wang X., Li C. (2024). Effect of *Zanthoxylum bungeanum* extract on the quality and cathepsin L activity of Niuganba. Meat Sci..

[5] Zhao S., Yang L., Hei M., Zhao Y., Zhu M., Wang H., Zhou H., Ma H. (2024). Conformation and functional modification of porcine myofibrillar protein by pepper leaf polyphenols under oxidative condition. LWT.

[6] Wang Z., He Z., Zhang D., Chen X., Li H. (2021). The effect of linalool, limonene and sabinene on the thermal stability and structure of rabbit meat myofibrillar protein under malondialdehyde-induced oxidative stress. LWT.

[7] Xu B., He W., Yi Y., Wang H., Xu W., Guo D. (2023). The impacting mechanism of β-sanshool in *Zanthoxylum bungeanum Maxim* on the structure of myofibrillar protein in duck meat. LWT.

[8] Wang J., Jiang W., Qiang Y., Han D., Huang F., Chisoro P., Jia W., Fauconnier M.L., Purcaro G., Zhang C. Deciphering Taste Endowment of *Zanthoxylum bungeanum* Maxim on Soy Sauce Marinated Beef: Binding of Key Taste Flavonols to Myofibrillar Protein. Available at SSRN 4828388. https://papers.ssrn.com/sol3/papers.cfm?abstract_id=4828388.

[9] Jiang J., Watowita P., Chen R., Shi Y., Geng J.-T., Takahashi K., Li L., Osako K. (2022). Multilayer gelatin/myofibrillar films containing clove essential oil: Properties, protein-phenolic interactions, and migration of active compounds. Food Packag. Shelf Life.

[10] Cai R., Duan M., Wang S., Lu F., Wu L., Tan Z., Zhang J., Shang P. (2021). Analysis on carcass performance and meat quality between the Tibetan pig and large white pig. J. Domest. Anim. Ecol..

[11] Gan M., Shen L., Fan Y., Guo Z., Liu B., Chen L., Tang G., Jiang Y., Li X., Zhang S. (2019). High altitude adaptability and meat quality in Tibetan pigs: A reference for local pork processing and genetic improvement. Animals.

[12] Wang S., Li J., Wang H., Han S., Guo C., Wang Y. (2013). A review on Tibetan swine (a)-carcass, meat quality, basic nutrition component, amino acids, fatty acids, inosine monophosphate and muscle fiber. Agric. Sci. Technol..

[13] Shen L., Lei H., Zhang S., Li X., Li M., Jiang X., Zhu K., Zhu L. (2014). The comparison of energy metabolism and meat quality among three pig breeds. Anim. Sci. J..

[14] Toldrá F. (2010). Handbook of Meat Processing.

[15] Poklar Ulrih N. (2017). Analytical techniques for the study of polyphenol–protein interactions. Crit. Rev. Food Sci. Nutr..

[16] Cheng J., Zhu M., Liu X. (2020). Insight into the conformational and functional properties of myofibrillar protein modified by mulberry polyphenols. Food Chem..

[17] Wang J., Vanga S.K., Raghavan V. (2019). High-intensity ultrasound processing of kiwifruit juice: Effects on the ascorbic acid, total phenolics, flavonoids and antioxidant capacity. LWT.

[18] Shi H., Li Y., Zheng J., Yao X., Wang W., Tomasevic I., Sun W. (2024). Effect of NaCl replacement by other salt mixtures on myofibrillar proteins: Underlining protein structure, gel formation, and chewing properties. J. Food Sci..

[19] Bae S.M., Jeong J.Y. (2024). Investigating the Effects of Pink-Generating Ligands on Enhancing Color Stability and Pigment Properties in Pork Sausage Model Systems Cured with Sodium Nitrite or White Kimchi Powder. Foods.

[20] Zhou Y., Yang M., Yin J., Huang J., Yan Y., Zhang F., Xie N. (2023). Physicochemical characteristics and gel-forming properties of mandarin fish (Siniperca chuatsi) protein during the fish fermentation with Lactobacillus sake SMF-L5: The formation of garlic-cloves shaped protein gel. Food Chem..

[21] Yi S., Li Q., Qiao C., Zhang C., Wang W., Xu Y., Mi H., Li X., Li J. (2020). Myofibrillar protein conformation enhance gel properties of mixed surimi gels with *Nemipterus virgatus* and *Hypophthalmichthys molitrix*. Food Hydrocoll..

[22] Yan B., Jiao X., Zhu H., Wang Q., Huang J., Zhao J., Cao H., Zhou W., Zhang W., Ye W. (2020). Chemical interactions involved in microwave heat-induced surimi gel fortified with fish oil and its formation mechanism. Food Hydrocoll..

[23] Meng L., Jiao X., Yan B., Huang J., Zhao J., Zhang H., Chen W., Fan D. (2021). Effect of fish mince size on physicochemical and gelling properties of silver carp (*Hypophthalmichthys molitrix*) surimi gel. LWT.

[24] Zhao X., Han G., Wen R., Xia X., Chen Q., Kong B. (2020). Influence of lard-based diacylglycerol on rheological and physicochemical properties of thermally induced gels of porcine myofibrillar protein at different NaCl concentrations. Food Res. Int..

[25] Liu X., Zhang T., Xue Y., Xue C. (2019). Changes of structural and physical properties of semi-gel from Alaska pollock surimi during 4 °C storage. Food Hydrocoll..

[26] Jing N., Wang M., Gao M., Zhong Z., Ma Y., Wei A. (2021). Color sensory characteristics, nutritional components and antioxidant capacity of *Zanthoxylum bungeanum Maxim.* as affected by different drying methods. Ind. Crops Prod..

[27] Wang Y., Wang X., Yang H. (2023). Gelation properties of silver carp (*Hypophthalmichthys molitrix*) surimi as affected by phenolic compounds in lotus root knot extract. Int. J. Food Prop..

[28] Djenane D., Khaled B.M., Ben Miri Y., Metahri M.S., Montañés L., Aider M., Ariño A. (2024). Improved Functionality, Quality, and Shelf Life of Merguez-Type Camel Sausage Fortified with Spirulina as a Natural Ingredient. Foods.

[29] Buamard N., Benjakul S. (2015). Improvement of gel properties of sardine (*Sardinella albella*) surimi using coconut husk extracts. Food Hydrocoll..

[30] Zhang J., Meng J., Yun X., Dong T. (2023). Effect of Artemisia sphaerocephala Krasch gum on the gel properties of myofibrillar protein and its application in cooked sheep sausage. Food Hydrocoll..

[31] Liu P., Wang S., Zhang H., Wang H., Kong B. (2019). Influence of glycated nitrosohaemoglobin prepared from porcine blood cell on physicochemical properties, microbial growth and flavour formation of Harbin dry sausages. Meat Sci..

[32] Ingenbosch K.N., Vieyto-Nuñez J.C., Ruiz-Blanco Y.B., Mayer C., Hoffmann-Jacobsen K., Sanchez-Garcia E. (2021). Effect of organic solvents on the structure and activity of a minimal lipase. J. Org. Chem..

[33] Ma J., Wang X., Li Q., Zhang L., Wang Z., Han L., Yu Q. (2021). Oxidation of myofibrillar protein and crosslinking behavior during processing of traditional air-dried yak (*Bos grunniens*) meat in relation to digestibility. LWT.

[34] Cao H., Jiao X., Fan D., Huang J., Zhao J., Yan B., Zhou W., Zhang H., Wang M. (2019). Microwave irradiation promotes aggregation behavior of myosin through conformation changes. Food Hydrocoll..

[35] Keppler J.K., Schwarz K., van der Goot A.J. (2020). Covalent modification of food proteins by plant-based ingredients (polyphenols and organosulphur compounds): A commonplace reaction with novel utilization potential. Trends Food Sci. Technol..

[36] Zhao Y., Wang W., Wu Y., Sun Q., Pan J., Dong X., Li S. (2025). Effects of *Eucommia ulmoides* Leaf Extract on the Technological Quality, Protein Oxidation, and Lipid Oxidation of Cooked Pork Sausage During Refrigerated Storage. Foods.

[37] Diao X., Zhu J., Huang L., Li S., Mao X., Li C., Ke W. (2024). Effect of citrus fiber on physicochemical quality of Frankfurter sausages: A study on lipid oxidation and protein gel characteristics. LWT.

[38] Jiang S., Ma Y., Wang Y., Wang R., Zeng M. (2022). Effect of κ-carrageenan on the gelation properties of oyster protein. Food Chem..

[39] Sharma S., Majumdar R.K., Mehta N.K. (2022). Gelling properties and microstructure of the silver carp surimi treated with pomegranate (*Punica granatum* L.) peel extract. J. Food Sci. Technol..

[40] Zhao S., Li M., Hei M., Zhao Y., Li J., Kang Z., Ma H., Xiong G. (2024). An Evaluation of the Effects of Pepper (*Zanthoxylum bungeanum Maxim.*) Leaf Extract on the Physiochemical Properties and Water Distribution of Chinese Cured Meat (Larou) During Storage. Foods.

[41] Xu Q.-D., Yu Z.-L., Zeng W.-C. (2021). Structural and functional modifications of myofibrillar protein by natural phenolic compounds and their application in pork meatball. Food Res. Int..

[42] Huang X., Sun L., Dong K., Wang G., Luo P., Tang D., Huang Q. (2022). Mulberry fruit powder enhanced the antioxidant capacity and gel properties of hammered minced beef: Oxidation degree, rheological, and structure. LWT.

[43] Cheng J.-R., Liu X.-M., Zhang Y.-S., Zhang Y.-H., Chen Z.-Y., Tang D.-B., Wang J.-Y. (2017). Protective effects of *Momordica grosvenori* extract against lipid and protein oxidation-induced damage in dried minced pork slices. Meat Sci..

[44] Chen B., Zhou K., Wang Y., Xie Y., Wang Z., Li P., Xu B. (2020). Insight into the mechanism of textural deterioration of myofibrillar protein gels at high temperature conditions. Food Chem..

[45] Zhao X., Han G., Sun Q., Liu H., Liu Q., Kong B. (2020). Influence of lard-based diacylglycerol on the rheological and physicochemical properties of thermally induced pork myofibrillar protein gels at different pH levels. LWT.

[46] Li J., Munir S., Yu X., Yin T., You J., Liu R., Xiong S., Hu Y. (2021). Double-crosslinked effect of TGase and EGCG on myofibrillar proteins gel based on physicochemical properties and molecular docking. Food Chem..

[47] Wasinnitiwong N., Benjakul S., Hong H. (2022). Effects of κ-carrageenan on gel quality of threadfin bream (*Nemipterus* spp.) surimi containing salted duck egg white powder. Int. J. Biol. Macromol..

[48] Huang X., Sun L., Liu L., Wang G., Luo P., Tang D., Huang Q. (2022). Study on the mechanism of mulberry polyphenols inhibiting oxidation of beef myofibrillar protein. Food Chem..

[49] Guan A., Mei K., Lv M., Lu J., Lou Q., Yang W. (2018). The effect of electron beam irradiation on IgG binding capacity and conformation of tropomyosin in shrimp. Food Chem..

[50] Zhao Y., Yuan Y., Yuan X., Zhao S., Kang Z., Zhu M., He H., Ma H. (2023). Physicochemical, conformational and functional changes of quinoa protein affected by high-pressure homogenization. LWT.

[51] Mi H., Li Y., Wang C., Yi S., Li X., Li J. (2021). The interaction of starch-gums and their effect on gel properties and protein conformation of silver carp surimi. Food Hydrocoll..

[52] Pan J., Lian H., Jia H., Hao R., Wang Y., Ju H., Li S., Dong X. (2020). Dose affected the role of gallic acid on mediating gelling properties of oxidatively stressed Japanese seerfish myofibrillar protein. LWT.

[53] Jiang D., Shen P., Pu Y., Jin M., Yu C., Qi H. (2020). Enhancement of gel properties of Scomberomorus niphonius myofibrillar protein using phlorotannin extracts under UVA irradiation. J. Food Sci..

[54] Fan M., Huang Q., Zhong S., Li X., Xiong S., Xie J., Yin T., Zhang B., Zhao S. (2019). Gel properties of myofibrillar protein as affected by gelatinization and retrogradation behaviors of modified starches with different crosslinking and acetylation degrees. Food Hydrocoll..

[55] Manzoor A., Haque A., Ahmad S., Hopkins D.L. (2023). Incorporation of betel leaf extract provides oxidative stability and improves phytochemical, textural, sensory and antimicrobial activities of buffalo meat sausages. Meat Sci..

[56] Anvari M., Chung D. (2016). Dynamic rheological and structural characterization of fish gelatin–Gum arabic coacervate gels cross-linked by tannic acid. Food Hydrocoll..

